# Dietary intake of vitamins B1, B2, B3, and B6, and the risk of gastric cancer: pooled analyses within the Stomach cancer Pooling (Stop) project

**DOI:** 10.1007/s00394-026-04112-6

**Published:** 2026-10-07

**Authors:** Giulia Collatuzzo, Claudio Pelucchi, Michele Sassano, Rossella Bonzi, Jesus Vioque, Sandra Gonzales-Palacios, Monireh Sadat Seyyedsalehi, Nuno Lunet, Samantha Morais, Gemma Castaño-Vinyals, Nerea Fernández Larrea-Baz, Monica Ferraroni, David Zaridze, Dmitry Maximovich, Guo-Pei Yu, Zuo-Feng Zhang, Lizbeth Lopez-Carrillo, Mary H. Ward, Malaquias Lopez-Cervantes, Raul Ulises Hernandez-Ramirez, Charles S. Rabkin, Reza Malekzadeh, Mohammadreza Pakseresht, Shoichiro Tsugane, Akihisa Hidaka, M. Constanza Camargo, Maria Paula Curado, Francesca Bravi, Eva Negri, Carlo La Vecchia, Paolo Boffetta

**Affiliations:** 1 Department of Biomedical and Clinical Sciences (DIBIC), Università degli Studi di Milano, Milano, Italy; 2 Branch of Medical Statistics, Biometry and Epidemiology ’G. A. Maccacaro’, Department of Clinical Sciences and Community Health, Dipartimento di Eccellenza 2023-2027, Università degli Studi di Milano, Milano, Italy; 3 Department of Medical and Surgical Science, University of Bologna, Bologna, Italy; 4 Instituto de Investigación Sanitaria y Biomédica de Alicante (ISABIAL), Alicante, Spain; 5 Centro de Investigación Biomédica en Red de Epidemiología y Salud Pública (CIBERESP), Madrid, Spain; 6 Unidad de Epidemiología de la Nutrición, Universidad Miguel Hernández (UMH), Alicante, Spain; 7 EPIUnit ITR, Instituto de Saúde Pública da Universidade do Porto, Universidade do Porto, Porto, Portugal; 8 Laboratório para a Investigação Integrativa e Translacional em Saúde Populacional (ITR), Porto, Portugal; 9 Departamento de Ciências da Saúde Pública e Forenses e Educação Médica, Faculdade de Medicina da Universidade do Porto, Porto, Portugal; 10 Universitat Pompeu Fabra (UPF), Barcelona, Spain; 11 ISGlobal, Barcelona, Spain; 12 National Center for Epidemiology, Carlos III Institute of Health, Madrid, Spain; 13 Fondazione IRCCS Ca’ Granda Ospedale Maggiore Policlinico, Milano, Italy; 14 Department of Clinical Epidemiology, N.N. Blokhin National Medical Research Center for Oncology, Moscow, Russia; 15 Medical Informatics Center, Pekin University, Pekin, China; 16 Department of Epidemiology, UCLA Fielding School of Public Health and Jonsson Comprehensive Cancer Center, Los Angeles, CA USA; 17 National Institute of Public Health, Morelos, Mexico; 18 Division of Cancer Epidemiology and Genetics, National Cancer Institute, National Institutes of Health, Rockville, MD USA; 19 Facultad de Medicina, UNAM, Coyoacán, Mexico; 20 Department of Biostatistics, Yale School of Public Health, Yale School of Medicine, New Haven, CT USA; 21 Digestive Oncology Research Center, Digestive Disese Reserach Institute, Tehran University of Medical Sciences, Tehran, Iran; 22 Department of Agricultural, Food and Nutritional Sciences, University of Alberta, Edmonton, AB Canada; 23 Division of Cohort Research, National Cancer Center Institute for Cancer Control, Tokyo, Japan; 24 International University of Health and Welfare Graduate School of Public Health, Tokyo, Japan; 25 Division of Epidemiology, National Cancer Center Institute for Cancer Control, Tokyo, Japan; 26 Centro Internacional de Pesquisa, A. C. Camargo Cancer Center, São Paulo, Brazil; 27 Stony Brook Cancer Center, Stony Brook University, Stony Brook, NY USA; 28 Department of Family, Population and Preventive Medicine, Renaissance School of Medicine, Stony Brook University, Stony Brook, NY USA

**Keywords:** Gastric cancer, Vitamins, Dietary intake, Micronutrients

## Abstract

**Background:**

Disentangling the role of micronutrients in gastric cancer (GC) is challenging and results are inconsistent to date. We investigated associations of dietary intake of B vitamins with GC in a pooled analysis of international studies.

**Methods:**

We included orifinal data from 13 case-control studies of GC from the Stomach Cancer Pooling Project. Data were harmonized and study-specific quartiles of dietary intake of the B1 (thiamine), B2 (riboflavin), B3 (niacin) and B6 (pyridoxine) were created. We calculated odds ratios (OR) of associations between each B vitamin with GC and the corresponding 95% confidence intervals (CI) through logistic regression models adjusted for major risk factors. We then pooled study-specific ORs using random effect meta-analysis. Stratified analyses were conducted by sex, histologic type and anatomical site. Secondary analyses were further adjusted for Helicobacter pylori (Hp) infection, alcohol, meat and vegetables and fruit intake.

**Results:**

The study population included 5252 GC cases and 11,397 controls. We observed the following associations for one-quartile increase of dietary vitamin intake: B1, OR = 0.93 (95% CI = 0.88–0.97); B2, OR=0.97 (95% CI = 0.93–1.02); B3, OR = 0.95 (95% CI = 0.90–1.00); B6, OR = 0.86 (95% CI = 0.82–0.91); we observed an interaction between B1 and sex, with beneficil effect in men for the highest quartile of intake (*p* = 0.009). Main results were confirmed when further adjusting for Hp. When accounting for alcohol, meat and vegetables and fruit intakes, the associations were weakened or no more apparent. No heterogeneity was found between cardia and non-cardia GC; B2 was directly associated with diffuse but not intestinal GC (*p* = 0.03).

**Conclusions:**

Dietary intake of B1 and B6 vitamins might reduce the risk of GC. Studies with homogeneous method of micronutrients assessment and adjustments are needed to confirm our results.

**Supplementary Information:**

The online version contains supplementary material available at https://doi.org/10.1007/s00394-026-04112-6.

## Introduction

Gastric cancer (GC) remains a major global health burden, despite being largely preventable [1]. Among risk factors for GC, besides *Helicobacter pylori (Hp)*, are certain dietary factors, such as consuming salt-preserved foods, red and processed meat and alcohol [2]. Information is inconsistent with regard to the role of micronutrients [3, 4]. Among these factors, vitamins play crucial roles in maintaining cellular functions and overall health [5, 6]. B vitamins represent an important dietary component [5, 6]. They include vitamin B1 (thiamine), B2 (riboflavin), B3 (niacin), B5 (pantothenic acid), B6 (pyridoxine), B7 (biotin), B9 (folate), and B12 (cobalamin). These micronutrients are essential to generate cofactors involved in enzymatic pathways which underlie vital cellular functions, such as favoring the metabolism of other nutrients, exerting an antioxidant effect, and supporting DNA repair [7]. As humans are unable to synthetize most B vitamins, they derive them mostly from diet [6]. The National Institute of Health Office of Dietary Supplements provides the recommended daily intake of B vitamins in the adult diet (B1: ∼1.1–1.2 mg, B2: ∼1.1–1.3 mg, B3: ∼15 mg, B5: ∼5 mg, B6: ∼1.3–1.7 mg, B7: ∼30 µg, B9: ∼400 µg and B12: ∼2.4 µg) [8].

The role of B vitamin in relation to the risk of GC has been investigated in several studies, however findings are inconsistent [9–22]. This can be in part explained by the large spectrum of cellular functions in which they are involved by differences in study design and analytical strategies [7].

The variety of dietary sources of these micronutrients make it challenging to disentangle their independent effects on GC [11].

The Stomach cancer Pooling (StoP) Project is an international consortium of studies on GC, with the objective of providing evidence-based epidemiological data [23]. Many of the participating studies were originally designed to investigate the relationship between GC and nutritional factors. A previous analysis based on data from the StoP Project showed an inverse association between folate intake and GC [19].

Our study aimed at investigating the role of dietary intake of vitamin B1, B2, B3, and B6, in the risk of GC, including its anatomical subsites and histological types, through a pooled analysis of international studies.

## Methods

The present study is based on the database version 3.3 of the StoP Project Consortium [23, 24], which includes 34 case-control studies, including several nested-within-cohorts, from 14 countries, for a total of 13,121 GC cases and 31,420 controls. The StoP Project aims at examining the role of several lifestyles and genetic determinants in the etiology of GC through pooled analyses of individual-level data, after central collection and validation of the original datasets. Participating studies were identified through bibliographic searches and personal contacts with the Principal Investigators. The Principal Investigators of each study provided a signed data transfer agreement and, thereafter, the de-identified data set of their respective studies. Detailed information on the overall aims, methods and main results of the StoP Project has been given elsewhere [23, 24].

The StoP Project was approved by the University of Milan Review Board (reference 19/15, April 1, 2015).

The present analysis was focused on vitamins B1, B2, B3, and B6 due to data availability and homogeneity of data across the participating StoP studies.

### Study population

Data on dietary vitamin B intake derived from 13 studies [25–37], as summarized in Supplementary Table 1. Three studies were from Mexico, two from Italy and Spain, and one each from Russia, Iran, China, Portugal, Spain, Japan and the USA, for a total of 5,252 cases and 11,397 controls. Data on vitamin B1 and B2 were available in 12 studies [25–31, 33–37], for a total of 4684 cases and 10,470 controls; data on vitamin B3 were available in 11 studies [25, 26, 28–31, 33–37], for a total of 4234 cases and 9,859 controls; and data on vitamin B6 were available in 11 studies (25, 27, 29–37), for a total of 3362 cases and 9060 controls. Cases and controls were matched in 11 out of 13 studies. The flow-chart is illustrated in Supplementary Fig. 1.

### Exposure assessment

Dietary intake information was collected in each study using semiquantitative food frequency questionnaires (FFQ), referring to the year prior GC diagnosis, in order to reduce the risk of reverse causation in reporting this information. Vitamin intake was calculated by each study using country-specific estimates of portion sizes and dietary composition tables. We did not include information on supplemental B vitamins intake because this information was available for a limited set of studies, with variable degree of detail on the type of supplementation and few data on the dose. Data were harmonized according to a pre-specified format, and completeness and consistency between variables were carefully checked. We first computed study-specific quartile categories. These study-specific quartiles were subsequently pooled into a single variable for each vitamin. Individuals reporting intakes of < 500 or > 4500 kcal/day were excluded. Additional study-specific information, including mean intake of specific B vitamin, is provided in Supplementary Table 1.

### Statistical analysis

Our main analysis consisted of calculating the overall odds ratios (ORs) and the corresponding 95% confidence intervals (CIs) through multivariate unconditional logistic regression. ORs were calculated for intake quartiles 2–4 compared to quartile 1, and for a linear increase per quartile. The main regression model (model 1) included study site, sex, age (continuous), tobacco smoking status (never, former, current smoker), socioeconomic status (low, intermediate, high as defined in each original study based on education, income or occupation) and total energy intake (quartiles; total number of missing data equal to 695). A sensitivity analysis was done by additionally adjusting model 1 for *Hp* infection, for studies in which the information was available (total number of missing data equal to 9161).

A second model (model 2) consisting of model 1 additionally adjusted for alcohol drinking [never, low ( < = 12 g/day), intermediate (12–47 g/day), high (> 47 g/day)], meat intake (quartiles: < 53 g/day; 53–88.3 g/day; 88.4–130 g/day; >=130 g/day ) and vegetable and fruit intake (low, intermediate, high – based on study-specific tertiles coded in the harmonized Stop database) was done. Additional models excluding from model 2 each of these three dietary factors one at a time were done to assess their potential confounding effect on the association between B vitamin and GC. In further models, one dietary factor at a time was excluded from the regression model to identify its potential confounding on the association under study. For these additional covariates, missings distributed as follows: *N* = 3,084 for alcohol drinking; *N* = 1,643 for vegetables and fruit intake; *N* = 2226 for meat intake. Given that dietary variables were not available in all the studies included in the pooled analysis, these additional models were run in subsets of studies with available information. These results were compared to those based on model 1, within the same subsets of studies. Correlations between each B vitamin and the additional dietary covariates were tested, and analyses based on model 1 with mutual adjustment of other vitamins B were also performed.

We also performed analyses stratified by sex, anatomical subsite (cardia vs. non-cardia GC), histological type (diffuse vs. intestinal GC), study setting (hospital-based and population-based) and geography (Europe, Americas and Asia) based on model 1. Heterogeneity between strata was tested using Cochran’s Q test [38]. Cases with undefined anatomical site or histological type at histopathology exam were excluded from the analyses.

We evaluated the linear increase across quartiles by treating the quartile variable as a continuous variable in the logistic regression model. This approach tests for a linear trend across the categories. Furthermore, we evaluated the linear increase per mg/day of dietary intake, using a continuous variable for each of the investigated vitamins. We have formally tested the difference in mean vitamins intake between cases and controls using a Student’s t-test.

We used a two-stage modeling approach [39]. In the first stage, we calculated the OR and 95% CI for the association one quartile increase of dietary intake of each vitamin B and GC in multivariable logistic regression models in each study. In the second stage, we computed pooled estimates using a DerSimonian and Laird random-effects model [40]. Heterogeneity between studies was tested using the Q test [38]. A two-tailed p-value < 0.05 was considered statistically significant. Heterogeneity level was assessed according to Higgins (low: ∼ 25%; moderate: ∼ 50%; high: ∼ 75%) [41]. Sensitivity analysis was conducted excluding a study consistently resulting an outlier [28], particularly for some specific associations.

Supplementary Figs. 2 and 3 presents the Directed Acyclic Graphs of the investigated associations.

All analyses were performed using STATA, version 16.1 (Stata Corp., College Station, TX, US) [42].

## Results

General characteristics of the study population are shown in Table 1. There were 5252 cases with available information, comprising of 495 (9.4%) cardia and 2968 (56.5%) non-cardia GC, and including 1159 (22.1%) diffuse-type and 1770 (33.7%) intestinal-type GC. Cases were mostly males (62.1%) and from low socioeconomic status (56.8%).

**Table 1:** Characteristics of the study population

Characteristics	Cases, *N* (%) *N* = 5252 (31.8)	Controls, *N* (%) *N* = 11,397 (68.2)
*Study (Ref)*		
Italy [25]	230 (4.4)	547 (4.8)
Italy, 4 areas [26]	1016 (19.4)	1159 (10.2)
Russia [27]	450 (8.6)	611 (5.4)
Iran [28]	286 (5.5)	304 (2.7)
China [29]	711 (13.5)	711 (6.2)
Portugal [30]	692 (13.2)	1667 (14.6)
Spain, 10 provinces [31]	441 (8.4)	3440 (30.2)
Spain [32]	401 (7.6)	455 (4.0)
Mexico [33]	248 (4.7)	478 (4.2)
Mexico [34]	220 (4.2)	752 (6.6)
Mexico [35]	234 (4.5)	468 (4.1)
Japan [36]	153 (2.9)	303 (2.7)
USA [37]	170 (3.2)	502 (4.4)
*Sex*		
Male	3,261 (62.1)	6233 (54.7)
Female	1991 (37.9)	5164 (45.3)
Mean age, years	62.9, 62.6–63.3	61.4, 61.1–61.6
Mean caloric intake Missing	2267.1, 2243.4-2290.8 *N* = 176	2132.3, 2117.8–2146.9 *N* = 519
*Socioeconomic status*		
Low	2981 (56.8)	5182 (45.5)
Intermediate	1651 (31.4)	3871 (34.0)
High	532 (10.1)	2203 (19.3)
Missing	88 (1.7)	141 (1.2)
*Tobacco smoking status*		
Never	2450 (46.7)	5498 (48.2)
Former	1311 (25.0)	2970 (26.1)
Current	1384 (26.3)	2730 (24.0)
Missing	107 (2.0)	199 (1.8)
*Subsite*		
Cardia	495 (9.4)	NA
Non-cardia	2968 (56.5)	
Undefined	817 (15.6)	
Missing	972 (18.5)	
*Histological type*		
Diffuse	1159 (22.1)	NA
Intestinal	1770 (33.7)	
Undefined	815 (15.5)	
Missing	1306 (24.9)	
*Dietary intake of vitamin B1-thiamine*		
Mean (mg/day), 95% CI	1.83, 1.75–1.92	3.23, 3.16–3.30
p	< 0.001	
*Dietary intake of vitamin B2-riboflavin*		
Mean (mg/day), 95% CI	1.83, 1.79–1.86	1.83, 1.81–1.85
p	0.71	
*Dietary intake of vitamin B3-niacin*		
Mean (mg/day), 95% CI	17.7, 17.5–17.9	18.4, 18.3–18.6
p	< 0.001	
*Dietary intake of vitamin B6-pyridoxine*		
Mean (mg/day), 95% CI	1.87, 1.77–1.98	1.94, 1.93–1.96
p	0.04	
Alcohol Never Low ( < = 12 g/day) Intermediate (12–47 g/day) High ((> 47 g/day) Missing	1001 (19.0) 865 (16.5) 1553 (29.6) 647 (12.3) 1186 (22.6)	2688 (23.6) 2962 (26.0) 2828 (24.8) 1021 (9.0) 1898 (16.6)
Meat < 53 g/day 53–88.3 g/day 88.4–130 g/day >=130 g/day Missing	1263 (24.1) 1113 (21.2) 1099 (20.9) 1050 (20.0) 727 (13.8)	3129 (27.5) 2775 (24.3) 2260 (19.8) 1734 (15.2) 1499 (13.2)
Vegetable-fruits Low Intermediate High Missing	1681 (32.0) 1561 (29.7) 1586 (30.2) 424 (8.1)	2963 (26.0) 3443 (30.2) 3772 (33.1) 1219 (10.7)

The StoP Project

N, number of observations.; Ref, reference study; mg/day, milligrams per day; CI, confidence interval

Table 2 reports the OR and 95% CI of the association between each B vitamin investigated and GC, overall and by sex. Different studies contributed to the exposure-specific pooled analysis. Cases had a lower mean intake of vitamin B1 than controls (1.83 mg/day vs. 3.23 mg/day, *p* < 0.001).

**Table 2:** Odds ratio and 95% confidence interval of the associations between B vitamins and gastric cancer, overall and by sex

Vitamin	OR*, 95% CI	Men	Women	*P* het* for sex
B1-thiamine (mg/day) (0–5.3) Q1 (5.30–7.1) Q2 (7.1–9.3) Q3 (9.3–100) Q4 Per quartile of intake	*N* = 14,677 (4518 cases) Ref 0.85, 0.76–0.95 0.80, 0.70–0.91 0.80, 0.69–0.93 0.93, 0.88–0.97	*N* = 8,405 (2837 cases) Ref 0.79, 0.67–0.92 0.74, 0.63–0.88 0.72, 0.59–0.87 0.91, 0.85–0.96	*N* = 6272 (1681 cases) Ref 0.94, 0.79–1.12 0.87, 0.71–1.07 0.96, 0.75–1.23 0.97, 0.90–1.06	*0.22*
Continuous variable	1.03, 1.00-1.06	1.03, 1.00-1.07	0.99, 0.93–1.06	*0.29*
B2-riboflavin (mg/day) (0–3.2) Q1 (3.2–4.1) Q2 (4.1-5.0) Q3 (5.0-17.1) Q4 Per quartile of intake	*N* = 14,677 (4518 cases) Ref 0.85, 0.76–0.95 0.84, 0.74–0.94 0.92, 0.80–1.05 0.97, 0.93–1.02	*N* = 8405 (2,837 cases) Ref 0.83, 0.72–0.96 0.88, 0.75–1.03 0.91, 0.76–1.08 0.98, 0.93–1.04	*N* = 6272 (1681 cases) Ref 0.89, 0.75–1.06 0.81, 0.67–0.98 1.00, 0.80–1.23 0.98, 0.92–1.05	*0.72*
Continuous variable	1.01, 1.01–1.01	1.03, 0.95–1.11	1.05, 0.96–1.14	*0.75*
B3-niacin (mg/day) (0–17.3) Q1 (17.3–21.3) Q2 (21.3–27.2) Q3 (27.2–97.6) Q4 Per quartile of intake	*N* = 13,630 (4,075 cases) Ref 1.03, 0.91–1.15 0.90, 0.79–1.03 0.88, 0.75–1.03 0.95, 0.90-1.00	*N* = 7,868 (2,590 cases) Ref 0.98, 0.83–1.14 0.81, 0.68–0.96 0.80, 0.66–0.99 0.92, 0.86–0.98	*N* = 5,762 (1,485 cases) Ref 1.11, 0.94–1.32 1.06, 0.86–1.30 1.00, 0.76–1.32 1.01, 0.93–1.10	*0.09*
Continuous variable	0.99, 0.98-1.00	0.99, 0.98-1.00	0.99, 0.97–1.01	*1.00*
B6-pyridoxine (mg/day) (0–1.8) Q1 (1.8–2.3) Q2 (2.3–2.8) Q3 (2.8–11.1) Q4 Per quartile of intake	*N* = 12,055 (3256 cases) Ref 0.87, 0.76-1.00 0.69, 0.59–0.81 0.66, 0.55–0.79 0.86, 0.82–0.91	*N* = 6743 (2,029 cases) Ref 0.86, 0.72–1.03 0.67, 0.54–0.82 0.64, 0.51–0.80 0.86, 0.79–0.92	*N* = 5312 (1227 cases) Ref 1.25, 1.03–1.51 1.35, 1.07–1.71 2.17, 1.62–2.89 0.89, 0.81–0.97	*0.57*
Continuous variable	0.83, 0.76–0.90	0.84, 0.76–0.94	0.81, 0.71–0.94	*0.69*

The StoP Project

OR, odds ratio; CI, confidence interval; N= number of observations; P het, p for heterogeneity (*calculated per quartile of intake); mg/day, milligrams per day; Q, quartile; Ref, reference category

Estimates from logistic regression model (model 1) adjusted for study, sex, age, socioeconomic status, cigarette smoking and total calorie intake

Included studies: B1-thiamine= [25–31, 33–37]; B2-riboflavin= [25–31, 33–37]; B3-niacin= [25, 26, 28–31, 33–37]; B6-pyridoxine= [25, 27, 28, 30–37]

The analysis for vitamin B1 (*N* = 14,677 total observations, including 4,518 cases) revealed an inverse association with GC, with OR of 0.93 (95% CI = 0.88–0.97) per quartile of intake (corresponding to approximately 2 mg/d), and a 20% reduced risk for intake of > 7 mg/day. The inverse association was stronger among men. When considering the continuous variable, increasing intake of vitamin B1 resulted positively associated with GC (OR = 1.03, 95% CI = 1.00–1.06 per mg/day), and in men (p-het = 0.29).

The analysis on vitamin B2 (*N* = 14,677 observations, including 4,518 cases, mean intake 1.83 mg/day in cases and controls, *p* = 0.71) showed a non-significant inverse association with GC (OR = 0.97, 95% CI = 0.93–1.02 per intake quartile). When stratifying by sex, no difference was observed (p-het = 0.72). The continuous analysis suggested a borderline positive association (OR = 1.01, 95% CI = 1.01–1.01).

A borderline inverse association was observed with vitamin B3 (*N* = 13,630 observations, 4,075 cases, mean intake 17.7 mg/day in cases and 18.4 mg/day in controls, *p* < 0.001, OR = 0.95, 95% CI = 0.90-1.00 per intake quartile). The inverse association was apparently stronger in men. The continuous analysis confirmed a borderline inverse association (OR = 0.99, 95% CI = 0.98-1.00).

Vitamin B6 (*N* = 12,055 observations, 3256 cases, mean intake 1.87 mg/day in cases and 1.94 mg/day in controls, *p* = 0.04) was inversely related to GC (OR = 0.86, 95% CI = 0.82–0.91 per intake quartile, corresponding to approximately 0.5 mg/day), with a 34% reduced risk in the comparison of the highest vs. the lowest quartile of intake (> 2.82 vs. < 1.82 mg/day; OR = 0.66, 95% CI = 0.55–0.79). The association was similar in men and women (p-het = 0.57). The inverse association was also observed when evaluating increasing mg/day of intake of B6 (OR = 0.83-95% CI = 0.76–0.90).

According to the two-stage approach (Supplementary Figs. 4–7), results of the main analysis were confirmed, showing a significant inverse association for B1 (OR = 0.87, 95% ci = 0.77–0.99) and B6 (OR = 0.88, 95% CI = 0.83–0.94). However, significant between-study heterogeneity was observed, ranging moderate to high according to Higgins. In particular, the I² statistics were 81.0% for vitamin B1 (*p* < 0.001), 68.2% for vitamin B2 (*p* < 0.001), 55.8% for vitamin B3 (*p* = 0.012), and 77.3% for vitamin B6 (*p* < 0.001). Sensitivity analyses excluding the Iranian study confirmed high heterogeneity for B1 (I²=76.8%, *p* < 0.001) and B2 (I²=63.2%, *p* = 0.002), and moderate heterogeneity for B3 (I²=48.5%, *p* = 0.042); the Iranian study contributed substantially to the observed between-study heterogeneity for vitamin B6 (I²=8.2%, *p* = 0.366), while the overall inverse relationship with GC was confirmed.

Table 3 reports results adjusted for *Hp* status in the subset of the study population with available information, and the effect in the same subset without *Hp* adjustment. *Hp* status did not appreciably impact on the associations (p-het: 1.00 for all exposures), which showed significant inverse association for all vitamins except for B2.

**Table 3:** Odds ratio and 95% confidence interval of the associations between B vitamins and gastric cancer, adjusted and unadjusted for Helicobacter pylori infection

Vitamin	OR, 95% CI		
	Hp adjusted	No Hp adjustment	*P*-het
B1-thiamine *N* = 6958 (1851 cases)	0.84, 0.78–0.91	0.84, 0.78–0.91	1.00
B2-riboflavin *N* = 6958 (1851 cases)	0.95, 0.88–1.01	0.95, 0.89–1.01	1.00
B3-niacin *N* = 6188 (1495 cases)	0.84, 0.77–0.92	0.84, 0.77–0.92	1.00
B6-pyridoxine *N* = 6958 (1851 cases)	0.84, 0.78–0.91	0.84, 0.78–0.91	1.00

The StoP Project.

OR, odds ratio; CI, confidence interval; N= number of observations; P-het, p for heterogeneity*, calculated per quartile of intake

Estimates from logistic regression model (model 1) adjusted for study, sex, age, socioeconomic status, cigarette smoking and total calorie intake

Included studies: [27, 28, 30–32, 34–37]

Table 4 reports results stratified by anatomical subsite and histological type of GC.

**Table 4:** Odds ratio and 95% confidence interval of the association between B vitamins and gastric cancer stratified by anatomical subsite and histological type, per quartile of intake

Vitamin	Anatomical subsite OR, 95% CI			Histological type OR, 95% CI		
	Cardia	Non-cardia	*P*-het	Diffuse	Intestinal	*P*-het
B1-thiamine	*N* = 8,288 (422 cases) 0.90, 0.80–1.02	*N* = 10,846 (2,516 cases) 0.90, 0.85–0.96	01.00	*N* = 9,478 (988 cases) 0.98, 0.89–1.06	*N* = 9,915 (1,425 cases) 0.93, 0.87–1.01	0.37
B2-riboflavin	*N* = 8,288(422 cases) 0.96, 0.86–1.07	*N* = 10,846 (2,516 cases) 0.97, 0.92–1.03	0.87	*N* = 9,478 (988 cases) 1.09, 1.00-1.17	*N* = 9,915 (1,425 cases) 0.97, 0.90–1.03	0.03
B3-niacin	*N* = 7,593 (331 cases) 0.93, 0.80–1.06	*N* = 10,043 (2,317 cases) 0.94, 0.89-1.00	0.89	*N* = 8,758 (872 cases) 0.99, 0.91–1.09	*N* = 9,192 (1,306 cases) 0.99, 0.91–1.07	1.00
B6-pyridoxine	*N* = 7,593 (387 cases) 0.79, 0.69–0.91	*N* = 9,553 (1,883 cases) 0.84, 0.78–0.90	0.44	*N* = 8,725 (895 cases) 0.84, 0.76–0.93	*N* = 9,017 (1,187 cases) 0.89, 0.81–0.97	0.40

The StoP Project

OR, odds ratio; CI, confidence interval; N= number of observations; P-het = p for heterogeneity among the categories (subsites and histological types)

Estimates from logistic regression model (model 1) adjusted for study, sex, age, socioeconomic status, cigarette smoking and total calories

For vitamin B1, the overall inverse association was confirmed for both GC subsites and histological types, which was significant selectively for non-cardia GC ; for vitamin B2, results by anatomical subsite did not show significant differences (*p* = 0.87), while those for histological type showed a borderline increased risk of diffuse-type GC (OR = 1.09, 95% CI = 1.00–1.17 per increase quartile) and no significant association with intestinal type, with significant heterogeneity (p-het = 0.03); vitamin B3 was not significantly associated with cardia and showed a borderline inverse association with non-cardia GC (p-het = 0.89), and was not significantly associated with diffuse-type or intestinal-type GC (p-het = 1.00); vitamin B6 was inversely related to both subsites (p-het = 0.44) and histological types of GC (p-het = 0.40).

Supplementary Tables 4 and 5 reports results by setting and geography. Vitamin B6 demonstrated the most robust protection against GC, particularly in European and Asian studies, while vitamin B1 showed significant protection exclusively in European, population-based studies. Vitamin B3 revealed a region-specific protective effect in Asias studies, whereas vitamin B2 consistently showed no association.

When we investigated the potential interaction by sex, a statistically significant interaction was observed exclusively for B1-thiamine (p for interaction = 0.009 in the highest quartile). Specifically, the inverse relationship of high vitamin B1 intake was robust among men but substantially attenuated among women. In contrast, no significant interactions by sex were found for the other vitamins investigated, indicating that their associations with GC risk do not vary significantly between men and women (Supplementary Table 6).

Results of model 2 analyses, adjusted and unadjusted for dietary factors in a subset of studies, are shown in Table 5. The association between vitamin B1 and GC was only mildly weakened (OR = 0.94, 0.88–1.00 per quartile of intake); the inverse association was corroborated when excluding dietary factors one at a time, except in the model excluding meat intake (OR = 0.98, 95% CI = 0.92–1.04). A positive association was suggested between vitamin B2 and GC (OR = 1.05, 1.00–1.11); this was revealed when meat was excluded. Vitamin B3 was no longer associated with GC (OR = 0.99, 95% CI = 0.92–1.06 per quartile of intake) in any combination of adjustments for dietary factors. Finally, vitamin B6 was no longer associated with GC (OR = 0.97, 95% CI = 0.90–1.04 per quartile of intake), except when excluding vegetables/fruit from the model (OR = 0.88, 95% CI = 0.82–0.94).

**Table 5:** Odds ratios and 95% confidence intervals of the association between B vitamins and gastric cancer, pooled analysis additionally adjusted for alcohol drinking, vegetable-fruits intake, and meat intake

Vitamin	Model 2 – OR, 95% CI	Model 1, additionally adjusted for meat and vegetable-fruits intake	Model 1, additionally adjusted for meat intake and alcohol consumption	Model 1, additionally adjusted for alcohol consumption and vegetable-fruits intake
B1-thiamine *N* = 10,938 (3,092 cases) Per quartile of intake	0.94, 0.88-1.00	0.93, 0.88–0.99	0.90, 0.85–0.96	0.98, 0.92–1.04
B2-riboflavin *N* = 10,938 (3,092 cases) Per quartile of intake	1.05, 1.00-1.11	1.04, 0.99–1.10	1.01, 0.96–1.07	1.06, 1.00-1.12
B3-niacin (*N* = 9,921, 2,666 cases) Per quartile of intake	0.99, 0.92–1.06	0.98, 0.91–1.05	0.94, 0.87-1.00	1.04, 0.98–1.11
B6-pyridoxine *N* = 9,715 (2,529 cases) Per quartile of intake	0.97, 0.90–1.04	0.97, 0.90–1.05	0.88, 0.82–0.94	0.99, 0.92–1.06

The Stop Project

OR, odds ratio; CI, confidence interval; Adj, adjustment; Q, quartile; N= number of observations; Ref, reference category

Model 1: adjusted for study, sex, age, socioeconomic status, cigarette smoking, and total calories

Model 2: adjusted for study, sex, age, socioeconomic status, cigarette smoking, total calories, alcohol drinking, vegetable-fruits intake, and meat intake

When we tested correlations of B vitamins with the additional covariates, correlation was observed with food items but not with alcohol (Supplementary Table 7).

Analyses based on model 1 with mutual adjustment of other vitamins B confirmed the protective effects of B1 and B6 against GC, while B2 and B3 were not associated (Supplementary Table 2).

## Discussion

We found inverse relationships between vitamins B1 and B6 and GC, which is overall consistent with previous literature [11, 18, 20, 21, 43]; for vitamins B3 and B6, the initial inverse relationships were not confirmed after adjustments for dietary factors. No association emerged between vitamin B2 and GC. No significant difference was evidenced by sex and anatomical subsite, while a potential increased risk was suggested between B2 and diffuse-type GC but not intestinal-type GC. When *Hp* was accounted for in a subset of studies, the main results were confirmed. The associations attenuated once the models accounted for alcohol, meat and vegetables and fruit intakes, whereas vitamin B1 appeared to have an independent inverse relationship with GC.

Previous results on the association between B vitamins and GC are inconclusive [9–23]. Differences in exposure assessment, study design and statistical approaches likely limit the comparability across available studies [11, 21].

While case-control studies, including studies from Italy [9], Korea [12], Iran [17], Belgium [18], and Vietnam [21] (ranging from ~ 200 to ~ 1200 cases), consistently reported protective dietary patterns [9, 17, 18, 20, 21], several prospective cohorts (encompassing up to ~ 500,000 participants) frequently yielded null results [15, 16, 21, 22, 23].

In particular, a case-control study conducted in Vietnam showed inverse associations of B1 and B6, but not B2, with GC [20]. When stratifying by anatomical site, the significant associations were confirmed for non-cardia GC only, while analysis for cardia GC was impaired by low number of cases [20].

Cohort studies showed less consistent results. Kweon et al. described an inverse association between B2 dietary intake and distal GC, limited to premenopausal women in a Chinese cohort [22]. Eussen et al. described inverse associations for both vitamins B2 and B6 adjusting for Hp and smoking, which were of similar magnitude of our results [43].

Mocellin et al. conducted a meta-analysis including case-control studies, cohort studies and randomized controlled trials summarizing evidence on B6 and cancer risk, including different types of exposures, distinguishing observational studies on dietary intake, circulating B6 levels and B6 supplementation [11]. Overall, higher dietary intake of B6 was inversely associated with cancer risk, with stronger effect for gastrointestinal cancers [11]. In particular, dietary intake of vitamin B6 was inversely related with GC risk (RR = 0.66, 95% CI = 0.57–0.76, based on analyses including 2,937 GC), with a dose-response relationship (RR = 0.90, 95% CI = 0.81–0.99 per mg/day) which we also observed in our study, while blood level was not [11]. Furthermore, evidence summarized for randomized controlled trials indicated that B6 supplementation was not associated with cancer risk [11]. Consequently, the authors argued that the benefit seen for dietary intake might simply reflect an overall healthy diet rich in other protective factors, rather than a direct causal effect of vitamin B6 alone.

 [11].

Similarly, a systematic-review and meta-analysis of 12 studies showed an overall negative association between vitamin B6 and GC (OR = 0.83, 95% CI = 0.73–0.95, *p* = 0.006) [21]. However, when stratified by study design, a significant association was observed only among case-control studies (OR = 0.68, 95% CI = 0.51–0.89), while no association was identified in cohort studies [21]. That meta-analysis combined studies based on circulating B6 level and dietary B6 intake [21], and pooled a nested case-control study [43] showing an inverse association between vitamin B6 and GC with traditional case-control studies, which may have contributed to heterogeneity in the results.

Anticancer properties of B6 vitamin have been described in mice, including reduced cell proliferation, oxidative stress, NO production and angiogenesis [51]. Vitamin B6 has been suggested to exert biological effects beyond its cohenzyme function, including antioxidant and carbonyl-scavenging properties, as previously described [52].

Our study suggested an inverse association between vitamin B1 and GC. This is consistent with previous epidemiological literature [18, 20]. In our study, we observed an apparent discrepancy between the continuous and quartile analyses for vitamin B1 intake. While the quartile analysis showed a significant protective trend, the continuous model yielded a non-significant or slightly increased risk. This pattern suggests a non-linear threshold relationship between dietary B1 intake and GC risk, rather than a strict linear dose-response relationship. Vitamin B1 is a co-enzyme for transketolase, which participates in glucose metabolism of malignant cells [48] and has been found to be upregulated in GC [49]. This aligns with the result observed when we considered continuous models, which in fact can be distorted by outliers in the upper tail of intake, where massive thiamine availability might favor cancer cell growth. Our results add interesting data regarding B1-thiamine and GC, where literature reports both cancer-promoting and anti-cancer properties of this vitamin [49, 50].

Interestingly, our stratified analysis revealed a significant interaction by sex exclusively for B1, with an inverse association among men that was substantially attenuated in women. In contrast, the role of B6-pyridoxine and B2-riboflavin appeared homogeneous by sex. An hypothesis is that, being B1-thiamin a critical cofactor for metabolic clearance in alcohol consumption, which is generally more prevalent and intense in men than in women, a high B1 intake exerts a stronger beneficial effect against gastric carcinogenesis specifically in individuals exposed to higher levels of alcohol-induced oxidative stress and mucosal damage. This is supported by the asymmetric distribution of alcohol consumption in our study population, where nearly half women were non-drinkers and a minimal fraction reported heavy consumption, while the large majority of men were moderate-to-heavy consumers. Additionally, residual confounding or a potential ceiling effect in women, who might already achieve protective thiamine thresholds through lower overall energy intake requirements, cannot be entirely ruled out.

We observed distinct variations in the association between vitamin B2 and GC histological subtypes, with a borderline increased risk selectively observed for the diffuse-type. To our knowledge, the available literature does not report results for dietary B2 intake stratified by GC histological classification, nor does it offer mechanistic hypotheses for this finding. It is well established, however, that intestinal and diffuse GCs arise through distinct molecular and etiopathological pathways. While intestinal-type GC follows the classic Correa’s multistep cascade driven by chronic inflammation and mucosal atrophy, diffuse-type GC is predominantly initiated by intrinsic epigenetic alterations—specifically, the hypermethylation and subsequent silencing of the *CDH1* (E-cadherin) promoter gene, which leads to a loss of cellular adhesion. Because vitamin B2 acts as a mandatory coenzyme in the one-carbon metabolism network, it directly modulates the synthesis of S-adenosylmethionine, the primary methyl donor for DNA methylation. Consequently, high riboflavin availability might hyper-activate the methylation machinery in susceptible individuals, facilitating the epigenetic silencing of critical tumor suppressor genes like *CDH1* and thereby promoting diffuse-type carcinogenesis. This specific epidemiological effect may also be significantly modified by genetic polymorphisms (such as *MTHFR* or *MTRR* variants) that are differentially expressed between diffuse and intestinal tumors [12]. Finally, the fact that this positive association was uniquely unmasked in our dietary models when meat intake was excluded highlights critical nutritional confounding, suggesting that the biological role of riboflavin heavily depends on its specific food matrices across different populations.

Exploring the effect of a single vitamin from that of other micronutrients remains challenging [11]. A possible strategy to address this issue is to account for food intake in the analyses. However, adjusting for food items can result in overadjustment, which usually leads to an underestimate of the effect of the micronutrients. This might in part explain the heterogeneity observed in the available literature. B vitamins have common food sources, including cereals, dairies, fresh fruits and vegetables, and meats, which makes it hard to define the role of a single component on a specific health outcome [5, 44]. In addition, certain foods reduce specific B vitamin absorption. Further, these vitamins may lose part of their properties during the cooking process, contributing to exposure misclassification in studies which do not account for these aspects of diet [5].

When we accounted for dietary items, effect estimates weakened, with only vitamin B1 little changes. This reduced effect can be the result of either overadjustment or confounding by other nutrients. Indeed, this attenuation is predictable due to the intrinsic collinearity between micronutrients and the whole foods from which they are derived, such as meat, fruits, and vegetables, which represent primary food matrices for B vitamins. In this respect, following the same approach adopted in our previous pooled analysis on dietary fiber, the risk estimates for B vitamins after such adjustment mainly reflect their independent biological effect, or the effect of B vitamins derived from alternative sources (e.g., whole grains).

In our study, we observed an apparent discrepancy between the continuous and quartile analyses for vitamin B1 intake. While the quartile analysis showed a significant protective trend, the continuous model yielded a non-significant or slightly increased risk. This pattern suggests a non-linear relationship between dietary B1 intake and GC risk. Furthermore, continuous variables are highly sensitive to extreme intake values (outliers) in the upper tail of the distribution, which can artificially distort the linear slope. Therefore, the categorical approach via quartiles provides a more robust and biologically plausible representation of the nutritional threshold effect in this population.

Vitamins B can also exert an effect on GC through the immune system, being involved in lymphocyte migration [7]. Anticancer properties of B6 vitamin have been described in mice, including reduced cell proliferation, oxidative stress, NO production and angiogenesis [51]. Vitamin B6 has been suggested to exert biological effects beyond its cohenzyme function, including antioxidant and carbonyl-scavenging properties, as previously described [52].

While we cannot exclude that dietary intake of B vitamins is a surrogate of a healthy diet [11], the observation that a residual protective effect remains apparent even after foods adjustment is informative, suggesting a protective role irrespective of the food source. Micronutrients may explain the protective effects observed for specific food items, which is consistent with the increased cancer risk documented among individuals with a low consumption of vitamin-rich fruit and vegetable.

 [45]. Different dietary patterns, and consequently the contribution of the different sources of each vitamin, may characterize the various populations included in this pooled analysis. Moreover, assessment methods of nutrients intakes may also vary [11, 21], potentially affecting our pooled analysis. These issues may result in residual confounding and limit the comparison of our results with previous studies, as the methods of adjustment were heterogeneous.

We observed significant between-study heterogenetiy in our two-stage analysis [41]. Our sensitivity analyses showed that the Iranian study [28] introduced significant heterogeneity ofr vitamin B6, wherease its exclusion did not fully account for the heterogeneity observed for the other vitamins. Potential sources of the remaining between-study heterogeneity include differences in study populations, geographical and contextual characteristics, recruitment settings, calendar periods, dietary assessment methods, as well as differences in the distribution of established GC risk factors.

When stratifying by study design, the protective effects of vitamins B1 and B6 remained robust and significant in population-based studies, whereas they lost significance in hospital-based settings, likely due to potential selection bias or smaller sample sizes. Furthermore, a geographic-specific pattern emerged. The geographic heterogeneity suggests that the impact of the investigated B-vitamins may be modulated by continent-specific dietary habits or distinct lifestyle factors, such as tobacco use and alcohol consumption, and residual confounding which may differ across the populations. While the multivariable models accounted for study center and lifestyle, stratified analyses provided a deeper insight into the specific associations observed.

Limitations should be acknowledged. A major limitation is the case-control design of the included studies. B vitamins intake can be affected by recall bias [53],which might be differential, as dietary habits may be reported differently by cases and controls. The case-control design of the studies and the hospital-based recruitment of the controls in some of them are potential sources of selection bias. Furthermore, the potential for reverse causation cannot be ruled out: it is possible that early symptoms might have led cases to change dietary habits prior to diagnosis, and thismay have had an impact on dietary intake assessment. To overcome the risk of reverse causality, the questionnaire referred to diet in the years before diagnosis; on the other hand, this might have increased the risk of recall bias. Another limitation is the fact that the analysis pooled studies which were based on different methodologies, including the use of different FFQs used for exposure assessment and country-specific databases, which likely contributed to heterogeneity. Moreover, while several adjustments were accounted for, residual confounding cannot be ruled out; in particular, smoking intensity may confound the observed effect. Another constraint is that we focused on dietary intake without biomarkers of nutritional status (such as serum vitamin levels or specific metabolic indicators). While dietary assessment tools are valuable for capturing long-term intake trends, they cannot account for individual variations in nutrient absorption and bioavailability. Future studies incorporating both dietary data and biological markers are warranted to confirm these findings. Further, in our study, only dietary intake of B vitamins was investigated, and not supplements nor food fortification. The identification of a beneficial role of such micronutrients derived from foods does not imply that the same effect would be seen through the intake of specific supplements [11, 46, 47]. Additionally, significant heterogeneity between-study was observed in our two-stage analysis; however, the direction of the associations was consistent across studies, supporting the overall solidity of our findings. Also, even if we accounted for many important covariates, residual confounding cannot be ruled out [41].

Last, the analyses were not corrected for multiple comparisons.

Our data were from 13 case-control studies, covering countries from Europe, North America, Latin America, and Asia. The large sample size allowed for analyses with high statistical power. Uniquely detailed epidemiological and clinicopathological information was available, including confounders. We consistently retrieved and harmonized data on dietary intake of B vitamins rather than mixed source of exposures. Next, we provided results by anatomical subsite and histological type of GC based on large number of observations. Additional stratified analyses were performed by major population characteristics, geography and study setting, allowing a comprehensive interpretation of the results. Internal validity of our results was assessed through sensitivity and interaction analyses. The results gained further insight from the secondary analyses adjusted for dietary factors.

## Conclusions

This study found an inverse relationship between vitamins B1and B6 and GC risk overall and by subtype; a moderate inverse relationship was also suggested for vitamin B3, while no clear association emerged for vitamin B2. We identified asignificant interaction between B1 and sex, with a beneficial effect observed exclusively in men for the highest quartile of intake. These findings underscore the importance of evaluating gender-specific metabolic pathways in nutritional cancer epidemiology. Given the various food sources and the correlations of the investigated exposures, it is however difficult to draw conclusive statements regarding any single compound, despite an overall beneficial role being confirmed for B1 vitamin independent from dietary factors.

## Supplementary Information

Below is the link to the electronic supplementary material.

Supplementary Material 1

## Data Availability

Deidentified data will be made available upon reasonable request.
