# Supplementary material for: Dietary intake of vitamins B1, B2, B3, and B6, and the risk of gastric cancer: pooled analyses within the Stomach cancer Pooling (Stop) project
Source: Eur J Nutr. 2026 Oct 7;65(8):272. doi: 10.1007/s00394-026-04112-6 (PMC13645984; doi:10.1007/s00394-026-04112-6)
Supplement: Supplementary file 1 [file 394_2026_4112_MOESM1_ESM.docx]

**Supplementary materials**

Supplementary Table 1. Characteristics of the included studies. The Stop Project.

Supplementary table 2. Distribution of cases and control for each exposure across the included studies. The Stop Project.

Supplementary Table 3. Results of the analysis with mutual adjustment vitamins B. The Stop Project.

Supplementary Table 4. Results of the analysis stratified by study setting. The Stop Project.

Supplementary Table 5. Results of the analysis stratified by geographic area. The Stop Project.

Supplementary table 6. Stratified interaction analysis of dietary vitamins B intake and gastric cancer risk by sex (reference: men). The Stop Project.

Supplementary table 7. Evaluation of collinearity across the investigated exposures and dietary factors.

Supplementary Figure 1. Flow-chart of the study population. The StoP Project.

Supplementary Figure 2. Directed Acyclic Graphs of the main model

Supplementary Figure 3. Directed Acyclic Graphs of the model additionally adjusted for dietary factors.

Supplementary Figure 3. Results of the meta-analysis of the studies with data on the association between B1-thiamine and gastric cancer. The Stop Project.

Supplementary Figure 4. Results of the meta-analysis of the studies with data on the association between B2-riboflavin and gastric cancer. The Stop Project.

Supplementary Figure 5. Results of the meta-analysis of the studies with data on the association between B3-niacin and gastric cancer. The Stop Project.

Supplementary Figure 6. Results of the meta-analysis of the studies with data on the association between B6-pyridoxine and gastric cancer. The Stop Project.

Supplementary table 1. Characteristics of the included studies. The Stop Project.

| **Country** | **Year of recruitment** | **Population** | **Matching** | **Vitamin data and mean intake mg/day, 95% CI** |
| --- | --- | --- | --- | --- |
| Milan, Italy (25) | 1997-2007 | Case-control, hospital-based | Yes | B1-thiamine: 0.85, 0.84-0.87  B2-riboflavin: 1.46, 1.43-1.49  B3-niacin: 17.5, 17.1-17.8  B6-pyridoxine: 1.81, 1.78-1.85 |
| 4 areas, Italy  (26) | 1985-1987 | Case-control, population-based | Yes | B1-thiamine: 1.01, 0.99-1.02  B2-riboflavin: 1.40, 1.37-1.42  B3-niacin: 14.6, 14.4-14.7 |
| Moscow, Russia  (27) | 1996-1997 | Case-control, hospital-based | No | B1-thiamine: 1.32, 1.30-1.35  B2-riboflavin: 4.21, 4.12-4.31  B6-pyridoxine: 2.27, 2.21-2.33 |
| Ardabil, Iran (28) | 2005-2007 | Case-control, population-based | No | B1-thiamine: 1.93, 1.87-1.99  B2-riboflavin: 1.85, 1.80-1.91  B3-niacin: 20.9, 20.3-21.6  B6-pyridoxine: 1.87, 0.82-1.92 |
| Shanghai, Qingdao, China (29) | 1991-1993 | Case-control, population-based | Yes | B1-thiamine: 0.37, 0.35-0.38  B2-riboflavin: 0.56, 0.53-0.59  B3-niacin: 12.5, 12.2-12.8 |
| Porto, Portugal (30) | 1999-2006 | Case-control, population-based | Yes | B1-thiamine: 1.75, 1.73-1.77  B2-riboflavin: 1.98, 1.95-2.01  B3-niacin: 22.1, 21.8-22.4  B6-pyridoxine: 2.08, 2.06-2.11 |
| 10 provinces, Spain (31) | 2008-2012 | Case-control, population-based | Yes | B1-thiamine: 7.86, 7.72-8.00  B2-riboflavin: 1.70, 1.68-1.72  B3-niacin: 18.1, 17.9-18.3  B6-pyridoxine:1.86, 1.84-1.88 |
| Valencia, Spain  (32) | 1995-1999 | Case-control, hospital-based | Yes | B6-pyridoxine: 1.16, 0.76-1.56 |
| Mexico City, Mexico (33) | 2004-2005 | Case-control, population-based | Yes | B1-thiamine: 1.75, 1.72-1.79  B2-riboflavin: 1.64, 1.59-1.69  B3-niacin: 17.0, 16.6-17.4  B6-pyridoxine: 1.63, 1.58-1.68 |
| Mexico City, Mexico (34) | 1989-1990 | Case-control, population-based | Yes | B1-thiamine: 1.79, 1.75-1.83  B2-riboflavin: 2.12, 2.06-2.18  B3-niacin: 19.6, 19.1-20.1  B6-pyridoxine: 2.16, 2.11-2.22 |
| 3 areas, Mexico (35) | 1994-1996 | Case-control, hospital-based | Yes | B1-thiamine: 2.10, 2.05-2.15  B2-riboflavin: 2.08, 2.02-2.13  B3-niacin: 21.7, 21.2-22.3  B6-pyridoxine: 2.35, 2.29-2.41 |
| Nagano, Japan (36) | 1998-2002 | Case-control, hospital-based | Yes | B1-thiamine: 1.26, 1.21-1.31  B2-riboflavin: 1.81, 1.73-1.88  B3-niacin: 20.1, 19.2-20.9  B6-pyridoxine: 1.90, 1.83-1.97 |
| Nebraska, USA (37) | 1988-1993 | Case-control, population-based | Yes | B1-thiamine: 1.46, 1.41-1.51  B2-riboflavin: 2.29, 2.20-2.37  B3-riboflavin: 22.2, 21.4-22.9  B6-pyridoxine: 1.84, 1.76-1.91 |

Supplementary Table 2. Distribution of cases and control for each exposure across the included studies. The Stop Project.

| Study | Vitamin B1 Controls | Vitamin B1 Cases | Vitamin B2 Controls | Vitamin B2 Cases | Vitamin B3 Controls | Vitamin B3 Cases | Vitamin B6 Controls | Vitamin B6 Cases |
| --- | --- | --- | --- | --- | --- | --- | --- | --- |
| Milan, Italy (25) | 70.40% (547) | 29.60% (230) | 70.40% (547) | 29.60% (230) | 70.40% (547) | 29.60% (230) | 70.40% (547) | 29.60% (230) |
| 4 areas, Italy  (26) | 53.29% (1,159) | 46.71% (1,016) | 53.29% (1,159) | 46.71% (1,016) | 53.29% (1,159) | 46.71% (1,016) | Missing | Missing |
| Moscow, Russia  (27) | 57.59% (611) | 42.41% (450) | 57.59% (611) | 42.41% (450) | Missing | Missing | 57.59% (611) | 42.41% (450) |
| Ardabil, Iran (28) | 51.70% (304) | 48.30% (284) | 51.70% (304) | 48.30% (284) | 51.70% (304) | 48.30% (284) | 51.70% (304) | 48.30% (284) |
| Shanghai, Qingdao, China (29) | 49.96% (706) | 50.04% (707) | 49.96% (706) | 50.04% (707) | 49.96% (706) | 50.04% (707) | Missing | Missing |
| Porto, Portugal (30) | 71.65% (1,600) | 28.35% (633) | 71.65% (1,600) | 28.35% (633) | 71.65% (1,600) | 28.35% (633) | 71.65% (1,600) | 28.35% (633) |
| 10 provinces, Spain (31) | 89.97% (3,040) | 10.03% (339) | 89.97% (3,040) | 10.03% (339) | 89.97% (3,040) | 10.03% (339) | 89.97% (3,040) | 10.03% (339) |
| Valencia, Spain  (32) | Missing | Missing | Missing | Missing | Missing | Missing | 53.15% (455) | 46.85% (401) |
| Mexico City, Mexico (33) | 65.84% (478) | 34.16% (248) | 65.84% (478) | 34.16% (248) | 65.84% (478) | 34.16% (248) | 65.84% (478) | 34.16% (248) |
| Mexico City, Mexico (34) | 77.37% (752) | 22.63% (220) | 77.37% (752) | 22.63% (220) | 77.37% (752) | 22.63% (220) | 77.37% (752) | 22.63% (220) |
| 3 areas, Mexico (35) | 66.67% (468) | 33.33% (234) | 66.67% (468) | 33.33% (234) | 66.67% (468) | 33.33% (234) | 66.67% (468) | 33.33% (234) |
| Nagano, Japan (36) | 66.45% (303) | 33.55% (153) | 66.45% (303) | 33.55% (153) | 66.45% (303) | 33.55% (153) | 66.45% (303) | 33.55% (153) |
| Nebraska, USA (37) | 74.70% (502) | 25.30% (170) | 74.70% (502) | 25.30% (170) | 74.70% (502) | 25.30% (170) | 74.70% (502) | 25.30% (170) |
| TOTAL | 69.09% (10,470) | 30.91% (4,684) | 69.09% (10,470) | 30.91% (4,684) | 69.96% (9,859) | 30.04% (4,234) | 72.94% (9,060) | 27.06% (3,362) |

Supplementary Table 3. Results of the analysis with mutual adjustment vitamins B. The Stop Project.

| Vitamin B  (N=10,065) | OR, 95% CI |
| --- | --- |
| B1 | 0.85, 0.79-0.91 |
| B2 | 1.06, 0.99-1.13 |
| B3 | 1.05, 0.97-1.15 |
| B6 | 0.86, 0.80-0.94 |

Supplementary Table 4. Results of the analysis stratified by study setting. The Stop Project.

| Exposure | Population-based Study | Hospital-based Study |
| --- | --- | --- |
| B1-Thiamine | 0.93 (95% CI: 0.88-0.98); N = 11738 | 0.92 (95% CI: 0.83-1.02); N = 2939 |
| B2-Riboflavin | 0.96 (95% CI: 0.92-1.01); N = 11738 | 1.02 (95% CI: 0.93-1.11); N = 2939 |
| B3-Niacin | 0.96 (95% CI: 0.90-1.01); N = 11738 | 0.91 (95% CI: 0.80-1.03); N = 1892 |
| B6-Pyridoxine | 0.84 (95% CI: 0.78-0.90); N = 8261 | 0.91 (95% CI: 0.83-1.00); N = 3794 |

Estimates from logistic regression model (model 1) adjusted for study, sex, age, socioeconomic status, cigarette smoking and total calories.

Supplementary Table 5. Results of the analysis stratified by geographic area. The Stop Project.

| Exposure | Europe | Americas | Asia |
| --- | --- | --- | --- |
| B1-Thiamine | 0.66 (95% CI: 0.59-0.75); N = 9399 | 0.91 (95% CI: 0.75-1.10); N = 2842 | 1.08 (95% CI: 0.87-1.34); N = 2436 |
| B2-Riboflavin | 1.01 (95% CI: 1.01-1.02); N = 9399 | 1.00 (95% CI: 0.99-1.01); N = 2842 | 1.00 (95% CI: 1.00-1.01); N = 2436 |
| B3-Niacin | 0.99 (95% CI: 0.92-1.06); N = 8352 | 1.05 (95% CI: 0.92-1.19); N = 2842 | 0.85 (95% CI: 0.76-0.95); N = 2436 |
| B6-Pyridoxine | 0.91 (95% CI: 0.85-0.98); N = 8179 | 0.94 (95% CI: 0.84-1.06); N = 2842 | 0.50 (95% CI: 0.41-0.61); N = 1034 |

Estimates from logistic regression model (model 1) adjusted for study, sex, age, socioeconomic status, cigarette smoking and total calories.

Supplementary table 6. Stratified interaction analysis of dietary vitamins B intake and gastric cancer risk by sex (reference: men). The Stop Project.

| Vitamin | Interaction quartile (women vs men) | OR | 95% CI | p-value |
| --- | --- | --- | --- | --- |
| B1-thiamine (N=14,677) | Quartile 2 | 1.22 | 0.99 - 1.51 | 0.065 |
|  | Quartile 3 | 1.18 | 0.95 - 1.46 | 0.138 |
|  | Quartile 4 | 1.34 | 1.08 - 1.67 | 0.009 |
| B2-riboflavin (N=14,677) | Quartile 2 | 1.13 | 0.91 - 1.40 | 0.260 |
|  | Quartile 3 | 0.98 | 0.79 - 1.22 | 0.853 |
|  | Quartile 4 | 1.20 | 0.96 - 1.48 | 0.103 |
| B3-niacin (N=13,630) | Quartile 2 | 1.05 | 0.84 - 1.31 | 0.673 |
|  | Quartile 3 | 1.15 | 0.92 - 1.44 | 0.234 |
|  | Quartile 4 | 1.07 | 0.85 - 1.36 | 0.559 |
| B6-pyridoxine (N=12,055) | Quartile 2 | 1.01 | 0.79 - 1.28 | 0.952 |
|  | Quartile 3 | 1.07 | 0.84 - 1.38 | 0.572 |
|  | Quartile 4 | 1.09 | 0.85 - 1.39 | 0.520 |

N, number, OR, odds ratio, CI, confidence interval

Supplementary table 7. Evaluation of collinearity across the investigated exposures and dietary factors.

| Vitamin B | Dietary Factor | Correlation Coefficient (r) | Observations (N) | p-value |
| --- | --- | --- | --- | --- |
| Thiamine | Vegetables and Fruit | 0.3672 | 14,143 | <0.001 |
|  | Meat | 0.3931 | 13,561 |  |
|  | Alcohol | 0.1293 | 12,670 |  |
| Riboflavin | Vegetables and Fruit | 0.3865 | 14,143 |  |
|  | Meat | 0.2621 | 13,561 |  |
|  | Alcohol | 0.0477 | 12,670 |  |
| Niacin | Vegetables and Fruit | 0.3663 | 13,092 |  |
|  | Meat | 0.4234 | 12,508 |  |
|  | Alcohol | 0.1367 | 11,643 |  |
| Vitamin B6 | Vegetables and Fruit | 0.5115 | 11,431 |  |
|  | Meat | 0.3471 | 10,869 |  |
|  | Alcohol | 0.1557 | 11,351 |  |
| Thiamine | Vegetables and Fruit | 0.3672 | 14,143 |  |
|  | Meat | 0.3931 | 13,561 |  |
|  | Alcohol | 0.1293 | 12,670 |  |

Supplementary Figure 1. Flow-chart of the study population. The StoP Project.

Note: GC, gastric cancer

Supplementary Figure 2. Directed Acyclic Graphs of the main model

Supplementary Figure 3. Directed Acyclic Graphs of the model additionally adjusted for dietary factors.

Supplementary Figure 3. Results of the meta-analysis of the studies with data on the association between B1-thiamine and gastric cancer. The Stop Project.

Estimates from logistic regression model (model 1) adjusted for study, sex, age, socioeconomic status, cigarette smoking and total calories.

Supplementary Figure 4. Results of the meta-analysis of the studies with data on the association between B2-riboflavin and gastric cancer. The Stop Project.

Estimates from logistic regression model (model 1) adjusted for study, sex, age, socioeconomic status, cigarette smoking and total calories.

Supplementary Figure 5. Results of the meta-analysis of the studies with data on the association between B3-niacin and gastric cancer. The Stop Project.

Estimates from logistic regression model (model 1) adjusted for study, sex, age, socioeconomic status, cigarette smoking and total calories.

Supplementary Figure 6. Results of the meta-analysis of the studies with data on the association between B6-pyridoxine and gastric cancer. The Stop Project.

Estimates from logistic regression model (model 1) adjusted for study, sex, age, socioeconomic status, cigarette smoking and total calories.

Supplementary Figure 7. Results of the meta-analysis of the studies with data on the association between B6-pyridoxine and gastric cancer. The Stop Project.

Estimates from logistic regression model (model 1) adjusted for study, sex, age, socioeconomic status, cigarette smoking and total calories.
